# XMRV Induces Cell Migration, Cytokine Expression and Tumor Angiogenesis: Are 22Rv1 Cells a Suitable Prostate Cancer Model?

**DOI:** 10.1371/journal.pone.0042321

**Published:** 2012-07-27

**Authors:** Kristin Stieler, Udo Schumacher, Andrea Kristina Horst, Nicole Fischer

**Affiliations:** 1 Institute for Microbiology and Virology, University Medical Center Eppendorf, Hamburg, Germany; 2 Department of Anatomy and Experimental Morphology, University Medical Center Eppendorf, Hamburg, Germany; 3 Institute for Clinical Chemistry, University Medical Center Eppendorf, Hamburg, Germany; Institut Pasteur Korea, Republic of Korea

## Abstract

22Rv1 is a common prostate cancer cell line used in xenograft mouse experiments as well as in vitro cell culture assays to study aspects of prostate cancer tumorigenesis. Recently, this cell line was shown to harbor multiple copies of a gammaretrovirus, called XMRV, integrated in its genome. While the original prostate cancer xenograft CWR22 is free of any retrovirus, subsequently generated cell lines 22Rv1 and CWR-R1, carry this virus and additionally shed infectious gammaretroviral particles in their supernatant. Although XMRV most likely was generated by recombination events in cell culture this virus has been demonstrated to infect human cells in vitro and 22Rv1 as well as CWR-R1 cells are now considered biosafety 2 reagents. Here, we demonstrate that 22Rv1 cells with reduced retroviral transcription show reduced tumor angiogenesis and increased necrosis of the primary tumor derived from xenografted cells in scid mice when compared to the parental cell line. The presence of XMRV transcripts significantly increases secretion of osteopontin (OPN), CXCL14, IL13 and TIMP2 in 22Rv1 cells. Furthermore, these data are supported by in vitro cell invasion and differentiation assays. Collectively, our data suggest that the presence of XMRV transcripts at least partially contributes to 22Rv1 characteristics observed in vitro and in vivo with regard to migration, invasion and tumor angiogenesis. We propose that data received with 22Rv1 cells or equivalent cells carrying xenotropic gammaretroviruses should be carefully controlled including other prostate cancer cell lines tested for viral sequences.

## Introduction

Prostate cancer (PC) is the most common type of cancer in men in Western societies with more than 350.000 newly diagnosed cancers and over 90.000 actual deaths per year, solely in Europe, thereby representing a serious socio-economical problem. Prostate cancer reflects a heterogeneous and multi stage disease which provides a challenge in developing suitable in vitro and in vivo models.

In vitro models rely on a few prostate cancer cell lines available [1] which are of epithelial origin: the most common cell lines used are LNCaP [2], PC3 [3], DU145 [4] and as a common xenograft model also 22Rv1 cells [5]. These cell lines served in the past, and are still commonly applied, as models for investigating tumor progression, invasion, metastasis, new therapeutic strategies as well as drug resistance. Transplanted into immunodeficient mice these cell lines produce tumors which are similar to the parental tumor [5]. Such in vivo xenograft models have been established using LNCaP cells, 22Rv1 or PC3 cells grafted in immunodeficient SCID, NUDE or NOD-SCID mice. In the absence of an ideal mouse model exhibiting hyperproliferation and hyperplasia in epithelial cells (Prostatic Intraepithelial Neoplasia, PIN), high-grade PIN (HGPIN), adenocarcinomas and invasive prostate carcinomas (mice naturally do not develop PC), xenograft mouse experiments using tissue slices or human prostate cancer cell lines are widely used.

22Rv1 is derived from a relapsed xenografted tumor CWR22 which has been serially transplanted in nude mice [5]. In 2009, 22Rv1 cells have been demonstrated to carry multiple integrated copies of the gammaretrovirus XMRV (xenotropic murine leukemia virus related virus); these cells produce high-titers of the virus in the culture supernatant [6]. Recent work provides evidence that two cell lines generated from a xenograft tumor CWR22, 22Rv1 (CWR22Rv1) and CWR-R1, produce infectious XMRV particles in their supernatant [7].

XMRV has been originally identified in prostate tissue from patients with familial prostate cancer [8]; subsequent work provided evidence of XMRV protein expression in up to 23% of all prostate cancer cases [9]. However, multiple studies failed to detect XMRV in prostate cancer samples using PCR or IHC methods [10], [11], [12], [13], [14], [15], [16], [17], [18], [19] Due to the lack of sequence variability of XMRV gene fragments in patients’ isolates compared to sequence variability identified in a XMRV positive cell line 22Rv1 it was postulated that XMRV might be a laboratory contaminant rather than a true exogenous human virus [20]. These data are strengthened by recent data of Paprotka and colleagues analysing different passages of CWR22 xenografts: XMRV is present in 22Rv1 cells and CWR-R1 cells, however, early passages of the CWR xenograft do not carry any detectable XMRV sequences. These data are in favour of a recombination event during passaging of xenograft CWR22, thereby generating XMRV [7].

22Rv1 cells are a commonly used preclinical model of prostate cancer [21], [22], [23]. Only recently, this cell line was classified as a biosafety level 2 cell line. This cell line produces high titers of xenotropic gammaretroviral particles which can infect human cells [6], [7]; inbred mice cells usually carry a mutation in the receptor of these viruses, called Xpr1, and are not permissive for this group of viruses. However, certain mouse cells (feral mice and some inbred strains carrying the appropriate receptor allele [24], [25]) can be infected with the virus. Caution for the interpretation of data solely resulting from 22Rv1 cells carrying the virus have been discussed earlier [26] however not directly addressed in in vivo or in vitro experiments.

In this current study we analysed the causal link between the gammaretrovirus XMRV and the transformed phenotype of 22Rv1 cells using in vitro assays commonly used to study cell proliferation, migration and differentiation by comparing 22Rv1 cells and 22Rv1 cells with reduced viral titers in xenograft mouse experiments, in vitro migration, invasion and tube formation assays. We provide evidence that the gammaretrovirus XMRV significantly contributes to tumorigenesis of 22Rv1 xenografts in mice. These observations are supported by in vitro results demonstrating differences in cytokine release in 22Rv1 cells infected with XMRV and 22Rv1 cells with reduced viral transcripts. Furthermore, we provide evidence that XMRV infection in prostate stromal fibroblasts significantly induces changes in cytokine release. We observe differences in cell migration of LNCaP cells when used in in vitro cell migration and invasion assays together with culture supernatant of stromal cells infected with XMRV; supernatant of cells infected with amphotropic gammaretroviruses or XMRV env pseudotyped virus like particles does not influence cell migration of LNCaP cells indicating that this affect is specific for XMRV and not dependent on receptor interaction or receptor signalling.

In summary, our results indicate that the transforming capacity of 22Rv1 cells is strongly dependent on the presence of XMRV. Therefore, results obtained in experiments using 22Rv1 cells with regard to prostate cancer tumorigenesis have to be validated in other virus negative prostate cancer cell lines.

## Results

The prostate epithelial cell line 22Rv1, derived from a human prostatic carcinoma xenografted in immunodeficient mice, contains several copies of the gammaretrovirus XMRV integrated in the host cell DNA. XMRV actively replicates in this cell line resulting in virus containing infectious supernatant [6], [7]. 22Rv1 cells have been used in xenograft mouse experiments in the past without knowing that an infectious virus is shedded by this cell line. Despite the fact that XMRV most likely is not a virus circulating in the human population we analyzed the contribution of this virus to cell line characteristics: cell migration and cytokine release as well as tumor progression in immunodeficient mice.

### Xenografted 22Rv1 Tumors in SCID Mice with Reduced Viral Transcripts are Highly Necrotic and Show Less Vessel Formation

**Figure 1:**
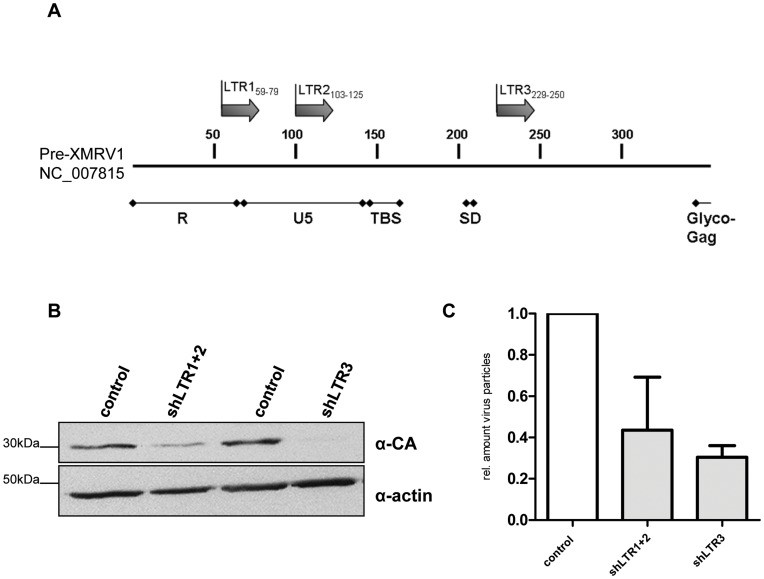
Stable knock down of XMRV in 22Rv1 cells. (**A**) Schematic representation of XMRV LTR region and 5′ UTR of Gag. Localization of shRNAs used to reduce XMRV transcripts in 22Rv1 cells is shown as arrows. Three different sequences, shLTR1 (R region), shLTR2 (U5 region) and shLTR3 (located shortly downstream of the splice donor site (SD)) were chosen using the Ambion’s siRNA Target Finder online tool. (**B**) 22Rv1 cells transduced with lentiviral supernatant containing the indicated shRNAs and puromycin selected: 22Rv1 shLTR1+2 cells show significantly reduced p30 protein (CA) in Western Blot analysis compared to control cells. Actin was used as a loading control. (**C**) Real-time PCR determining the relative amount of infectious particles in the supernatant of shRNA transduced 22Rv1 cells.

**Figure 2:**
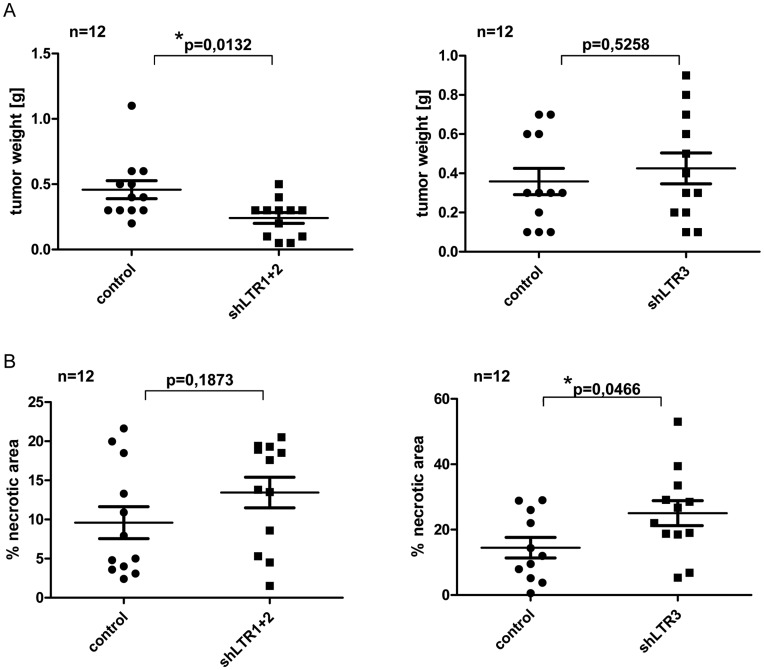
XMRV knock down reduces tumorigenicity of 22Rv1 *in vivo*. SCID mice (n = 6) were s.c. injected with 22Rv1 shLTR1+2, shLTR3 or with 22Rv1 control cells. For each cell line the final tumor amount was n = 12. 36d p.i. mice were sacrificed and tumors were analyzed for weight (A) and necrosis (B).

**Figure 3:**
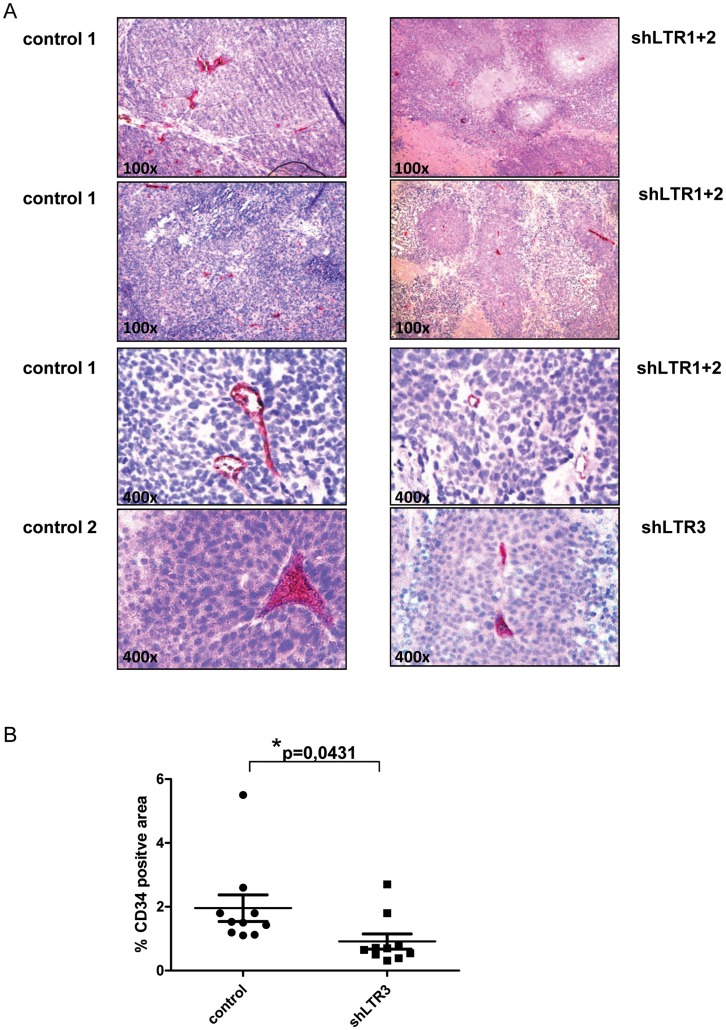
Vessel formation in xenografted tumors dependent on XMRV expression. (**A**) Immunohistochemistry staining for CD34 revealed rudimentary blood vessel formation in XMRV knock down tumors, while tumors of 22Rv1 control display emerged structures of angiogenesis. (**B**) CD34^+^ areas in tumors were quantified in control and shLTR3 tumors (n = 10 each). Percentages of CD34^+^ areas are expressed relative to the total areas analyzed.

We established a 22Rv1 cell line with reduced XMRV transcript amounts resulting in less infectious viral particles in the culture supernatant. Two different shRNAs targeting two different regions in the XMRV LTR region (Figure 1A) were combined. Stable hairpins containing the sequences shLTR1 and shLTR2 (Table S1) were individually cloned into the lentiviral vector LeGO G-puro [27]. Pseudotyped viral particles containing supernatant of both shRNAs containing lentiviral RNAs was used for the infection of 22Rv1 cells, which were subsequently treated with puromycin to select shRNA expressing cells. To rule out specific selection of individual integration events, bulk selection instead of single clone selection was performed as well as control supernatant containing pseudotyped viral particles with the parental lentiviral plasmid LeGo G-puro without shRNA insert was generated. As shown in Figure 1B Gag p30/CA (capsid) protein expression levels were significantly reduced as well as the amount of infectious particles shed into the supernatant was considerably decreased in shLTR1+2 expressing 22Rv1 cells (Figure 1C). Using these cells in xenograft in vivo experiments, a total of six SCID mice per shRNA group were subcutaneously injected with 2×10^6^ cells in matrigel in each lateral flank. Tumor onset and weight was monitored for 36d. We did not observe significant differences in the onset of tumor growth between 22Rv1 control cells and 22Rv1 cells expressing shLTR1+2 as judged by daily visual inspection and weight control of the mice (data not shown). After 36d, mice were sacrificed and tumor weight, necrosis as well as vessel formation was analyzed. The weight of 22Rv1 shLTR1+2 tumors was significantly reduced (Figure 2A left panel) compared to control tumors. These tumors displayed large necrotic areas (as seen in Figure 2B left panel, Figure S2A (control cells) and S2B (22Rv1 shLTR1+2)) and showed significantly fewer vessel numbers when performing immunohistochemical staining applying a CD34 monoclonal antibody to tumor tissue sections (Figure 3A upper panels and Figure 3B) as compared to the controls.

To rule out that the observed differences are a consequence of so called off target effects of shRNAs targeting XMRV transcripts or clonal selection of an individual integration event, a third shRNA with complementary sequences to the 5′GAG region (229–250, NC_007815) (Figure 1A) was cloned into LeGO G-puro plasmid. Similar to the 22Rv1 cells transfected with shLTR1+2, 22Rv1 cells expressing shLTR3, showed significantly reduced p30 CA/gag protein expression levels (Figure 1B, lane 3 and 4) and up to 80% less infectious viral particles in the culture supernatant (Figure 1C) compared to control cells. Injecting these cells into each flank of six SCID mice did not reduce the time point of tumor growth as well as there was no significant change in the weight of the tumor as observed for shLTR1+2 (Figure 2A, right panel). However, we observed large necrotic areas in 22Rv1 shLTR3 tumors (Figure 2B, right panel). While tumors of 22Rv1 shLTR1+2 showed only minor differences in the size of necrotic areas, these differences were statistically significant in tumors induced with 22Rv1 shLTR3 cells. Similarly, CD34 staining demonstrated decreased vessel formation based on CD34 positive IHC staining in tumors induced by 22Rv1 shLTR3 cells as compared to control tumors (Figure 3).

These experiments indicate that characteristics of 22Rv1 xenografts in immunodeficient mice partially depend on the production of the gammaretrovirus XMRV in these cells.

### 22Rv1 Cells Expressing shLTR1+2 Differ in Cytokine Expression Pattern Compared to Parental Cells

Culture supernatants from 22Rv1 control cells, infected with pseudotyped lentiviral supernatant containing the empty LeGO G puro plasmid and from 22Rv1 shLTR1+2 cells were collected and assayed for cytokines by using a solid-phase immunoblotting procedure (RayBio Human Cytokine Antibody Array 5; RayBiotech) (Figure S3). The supernatants were applied to membranes containing antibodies to 80 human cytokines. Minor differences in the release of cytokines from these cells were detected (Figure S3). In 22Rv1 cells with reduced XMRV transcript levels, Osteopontin (OPN), tissue inhibitor of matrixmetalloproteinase TIMP2 and IL13 were slightly reduced while hepatocyte growth factor (HGF) was increased.

**Figure 4:**
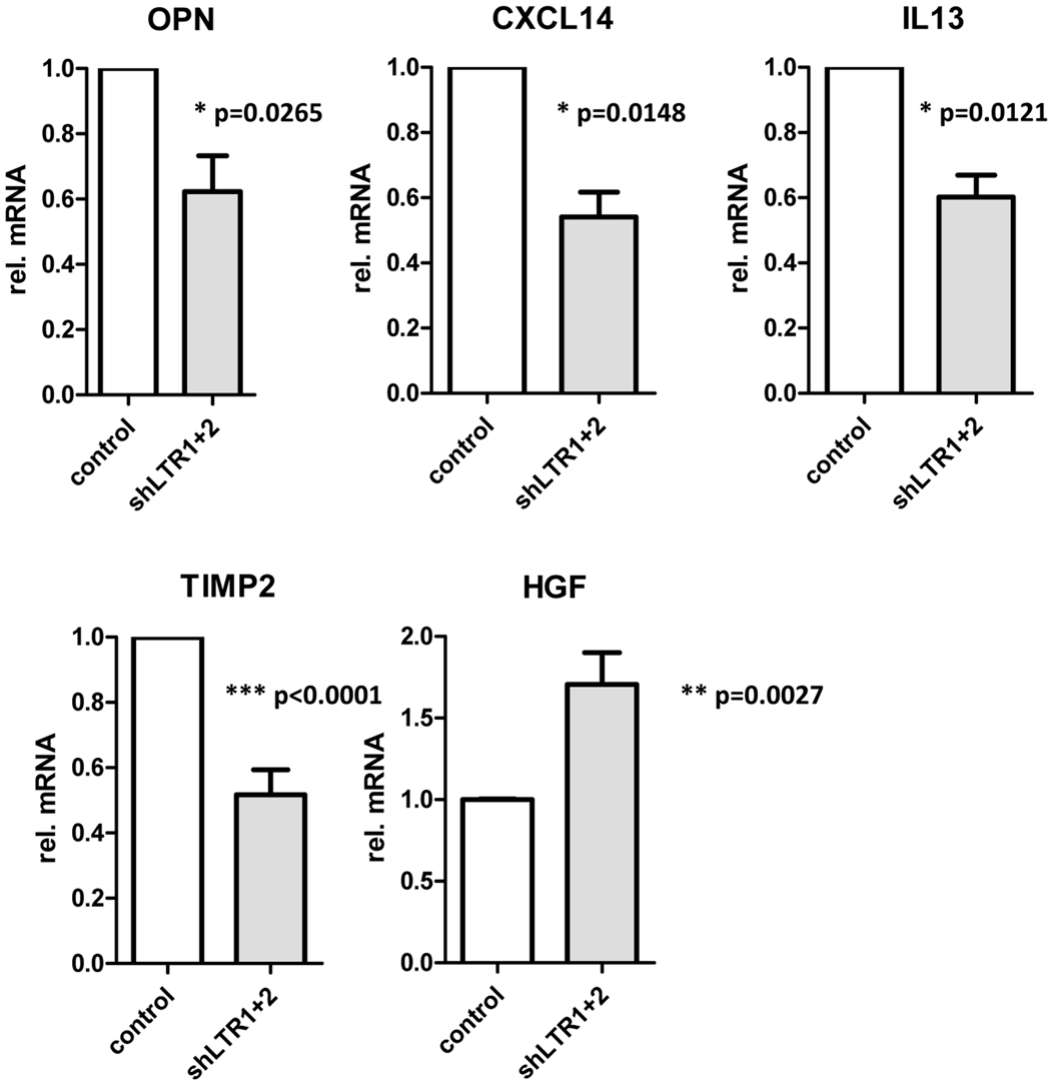
Differences in cytokine expression pattern in 22Rv1 cells dependent on XMRV expression. Total RNA of 22Rv1 control and 22Rv1 shLTR1+2 was analyzed for mRNA expression levels of the indicated cytokines by qRT-PCR. Data were normalized against three housekeeping genes (GAPDH, TBP, RLP13).

Using quantitative real-time PCR for the indicated mRNA transcripts we confirmed these findings: OPN, TIMP2 and IL13 expression is significantly reduced in 22Rv1 cells expressing shLTR1+2 while HGF expression is considerably increased (Figure 4). Additionally, we tested the expression of matrix metalloproteinase MMP9 and CXCL14 expression in 22Rv1 cells and 22Rv1 shLTR1+2 cells. While we did not find any differences in MMP9 mRNA expression in these cell lines (data not shown), CXCL14 expression was significantly reduced in the 22Rv1 shLTR1+2 cells.

### De Novo Infection of Prostate Stromal Fibroblast with Replication Competent XMRV Induced Differences in Cytokine Expression

To analyze whether the observed differences in cytokine expression and release is cell type dependent, we analyzed cytokine expression and release in prostate stromal fibroblasts (PrSc) infected with replication competent XMRV derived from LNCaP cells transfected with XMRV VP62 proviral DNA. PrSc were isolated from prostate tissue by collagenase digest and culturing in selective media as described recently [28], [29]. Outgrowing cells were expanded and confirmed by FACS staining for the expression of α-SMA (smooth muscle actin) and vimentin (markers for activated stromal fibroblast) and lack of cytokeratin expression [28]. Only low passage numbers of these cells were used in XMRV infection experiments. 2d past infection supernatant of XMRV infected PrSc or mock infected cells was applied to a commercial antibody membrane arrays (RayBio; see Figure S4). XMRV infection of the cells was confirmed by XMRV gag specific PCR (data not shown). Of the 60 cytokines analyzed by antibody membrane arrays eight (GRO, GROα, TIMP1, TIMP2; HGF, IGFBP2, IGFBP4, IL13) were differentially expressed by XMRV infected compared to MOCK infected cells. While GRO and GROα transcripts were up regulated in supernatant from PrSC XMRV infected cells, HGF, IGFBP2, IGFBP4, IL13, TIMP1 and TIMP2 expression was reduced.

**Figure 5:**
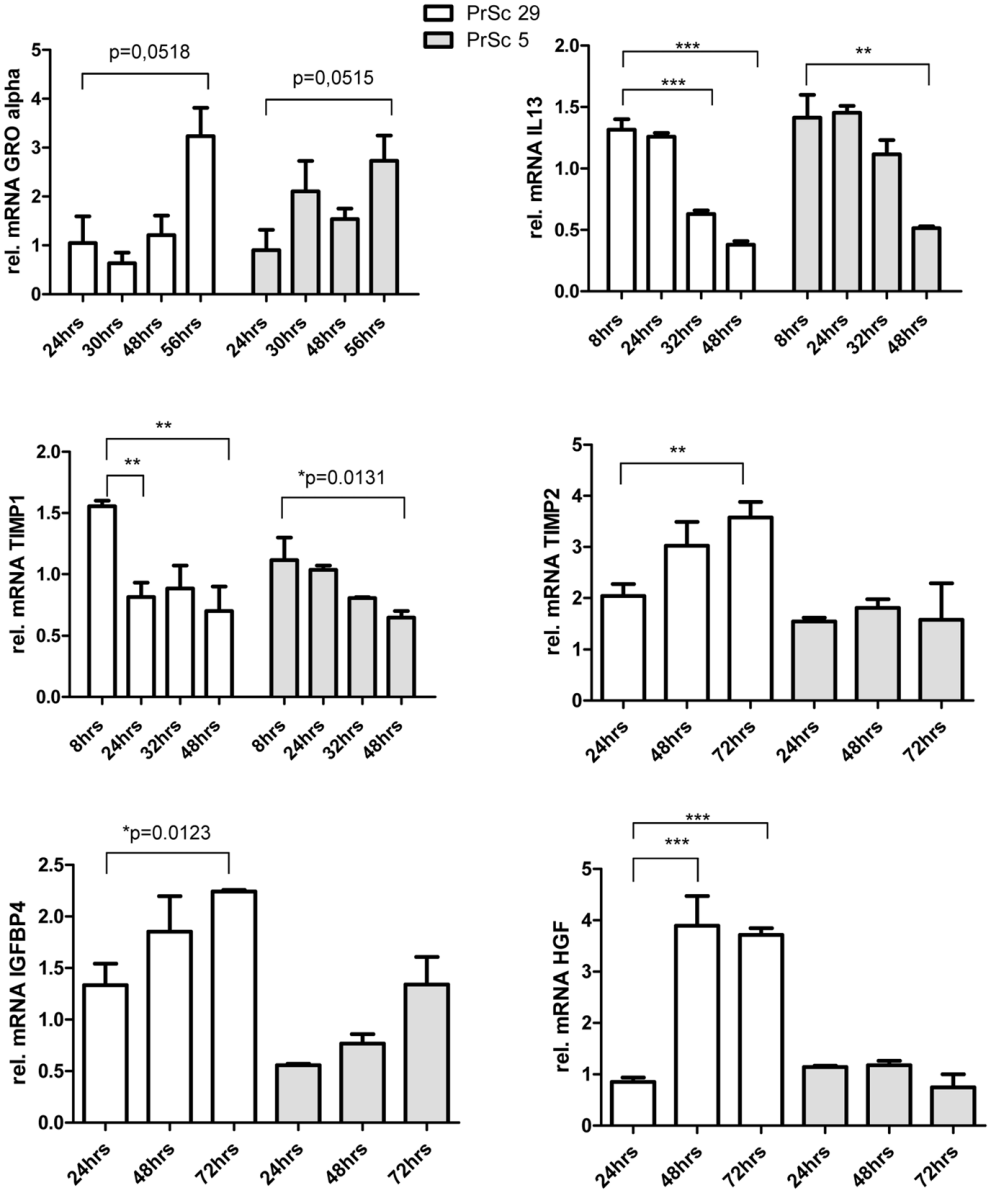
Differences in cytokine expression of primary prostate stromal fibroblasts infected with XMRV. Primary prostate stromal fibroblasts (PrSc) were infected with XMRV containing supernatant of LNCaPi cells or with mock supernatant. Total RNA was analyzed for mRNA expression levels of different cytokines by qRT-PCR. Data were normalized against three different housekeeping genes and illustrated as relative gene expression compared to mock infected cells at each individual time point.

To confirm the results obtained by cytokine antibody arrays and to exclude differences in cell preparation of primary prostate stromal fibroblasts, we performed quantitative real-time PCR experiments using two different PrSc stromal fibroblast lines obtained from different patients (Figure 5). Cells were infected with culture supernatant containing replication competent XMRV derived from LNCaP cells transfected with XMRV VP62 proviral DNA [30], [31]; total RNA was extracted, DNaseI treated and subsequently analyzed by qRT-PCR for differences in cytokine expression. While we were able to confirm our results obtained by Ab array for GROα, IL13 and TIMP1, we were unable to confirm our preliminary data for TIMP2, HGF and IGFBP4. TIMP2 and IGFBP4 expression increased in response to XMRV infection and induction of HGF expression was only observed in PrSc29 cells, while PrSc5 cells showed the opposite effect.

### Cytokine Release Dependent on XMRV Replication Influences Invasiveness and Tube Formation in vitro

**Figure 6:**
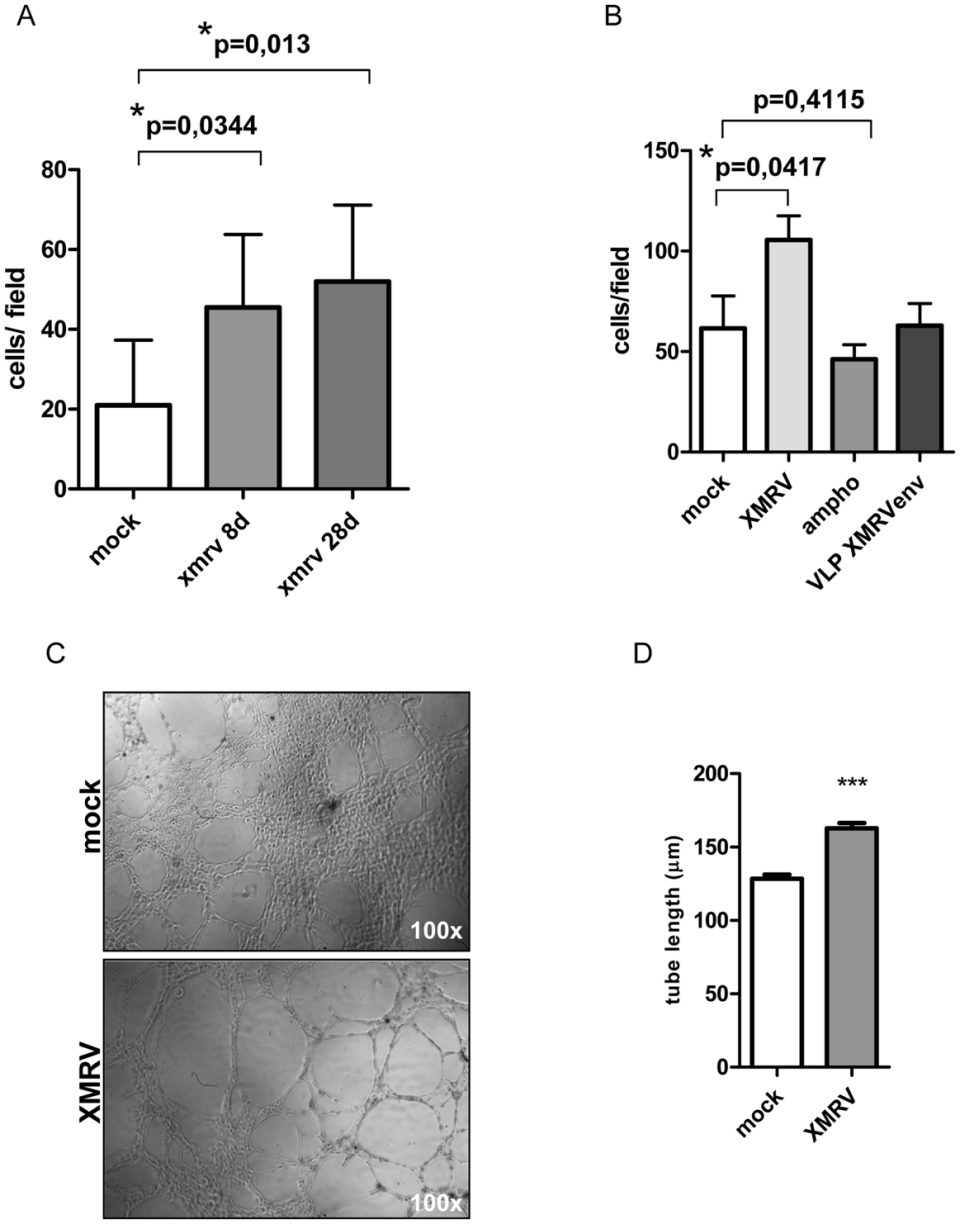
Cytokine release of XMRV infected stromal cells increases invasiveness of prostate cancer cells *in vitro*. (A) Primary stromal fibroblasts (PrSc) 8d or 28d p.i. with XMRV were seeded in the bottom compartment of an invasion chamber. Invasiveness of LNCaP cells was calculated in three independent experiments performed in duplicates. (B) PrSc cells infected with xenotropic MLVs (XMRV) induce invasion of prostate cancer cells in vitro while stromal cells infected with amphotropic MoMCF or XMRV env pseudotyped virus like particles do not increase invasion of LNCaP cells. (C) and (D) XMRV infection induces tube formation of HMEC cells. Cells seeded on matrigel were incubated with supernatant of either XMRV or mock infected stromal fibroblasts and tube formation was followed for 5 h. Representative picture of tube formation observed is shown in (C). (D) Quantification of two independent experiments performed in triplicates. Pictures of three different visual fields per well were analyzed for tube length (20 tubes per field) using the Adobe Photoshop ruler tool.

To obtain a mechanistic understanding of the observed differences in cytokine release of prostate epithelial or stromal cells infected with XMRV, several in vitro studies were performed. We did not observe differences in cell proliferation between infected (22Rv1 control shRNA and 22Rv1 shLTR1+2 or PrSc infected and non-infected PrSc cells applying an MTT assay (Figure S1). Next, we investigated whether XMRV infected cells could promote the migration of LNCaP cells in a paracrine manner. Primary prostate stromal fibroblasts either mock or XMRV infected were seeded in the bottom compartment of a 24-transwell chamber dish. Through a permissive membrane released cytokines directly affect invasiveness of LNCaP cells. Using this assay we performed a course on an XMRV infection, shown in Figure 6A. XMRV infection of stromal fibroblast results in statistically significant increase of migrating LNCaP cells which is furthermore increased in cells infected for longer period of time (28d). This increase is specific for XMRV since a related MLV (MoMCF, an amphotropic MLV using PIT2 as receptor) did not result in higher amounts of migrating LNCaP cells (Figure 6B). Interestingly, replication incompetent virus like particles pseudotyped with XMRV env did not result in an increase of LNCaP cell migration (Figure 6B) suggesting that events independent of receptor binding and signaling contribute to the observed phenotype.

Additionally, we performed in vitro angiogenesis assays: tube formation was analyzed using supernatants from either XMRV or MOCK infected cells as a stimulant as they contained differentially expressed/or different levels of pro-angiogenic cytokines. Culture supernatant from prostate stromal fibroblasts was added to HMEC cells on matrigel and tube formation was followed for 5 hrs. Changes in cytokine release in response to XMRV infection significantly increased the length of the capillary network formed by HMEC cells (Figure 6C and D), which is in line with our in vivo findings.

## Discussion

Our study demonstrates that xenotropic gammaretroviruses, which have been frequently identified in cancer cell lines, established by passing those though nude mice, not only bear the risk of infection but also could significantly influence the experimental data received with these cell lines. In particular, the prostate cancer cell line 22Rv1 commonly used in prostate cancer in vitro studies as well as in vivo xenograft models carries multiple copies of XMRV integrated and actively sheds infectious virus in its supernatant.

XMRV was originally identified in human prostate cancer tissue and its association with human pathologies has been suggested in the last years. Recent data suggesting a recombination of two ancestor mouse XMRV sequences, called Pre-XMRV1 and Pre-XMRV2, in cell culture thereby generating XMRV [7] together with multiple studies not detecting XMRV sequences in human samples [11], [12], [14], [17], [18], [20], [32], [33], [34], [35], [36], [37] question an association of XMRV with human diseases.

Still, the analysis of XMRV transformation potential is of significant interest since 22Rv1 cells and CWR22-R1 cells produce infectious virus and are a generally accepted in vitro model as well as xenograft model to study prostate cancer tumorigenesis, progression, biomarkers and the development of therapeutical strategies.

In this current study we generated a 22Rv1 cell line with reduced XMRV transcripts and viral protein levels by the application of shRNAs targeting two different regions in the XMRV LTR region. in vivo experiments showed that tumors induced by 22Rv1 cells with reduced XMRV transcripts were highly necrotic and showed significantly less vascularization as judged by CD34 staining (Figure 3) and CEACAM-1 staining (data not shown). To exclude integration site selection as well as shRNA off target affects these experiments were repeated using independent 22Rv1 LeGO-shXMRV LTR infection experiments as well as using different shRNA sequences targeting a third region in XMRV (shLTR3). In contrast to the experiments using 22Rv1 shLTR1+2 cells, no significant differences in tumor size were observed which might be due to the small number of animals per group. Alternatively, the differences observed in tumor size in the first set of xenograft experiments using shRNA sequences targeting the LTR regions 59–79 and 103–125 could be a consequence of so called off-target effects in the cells. We point out that instead of shRNA against luciferase or other sequences generally not found in the human genome, an empty lentiviral vector was used to generate control lentiviral supernatant subsequently used to transduce 22Rv1 cells. Thus, we cannot exclude a saturation of components of the siRNA machinery resulting in unspecific effects. However, we did not observe any phenotype, increased levels of apoptosis, reduced viability of cells, that has been described to be correlated with unspecific effects caused by saturating the siRNA pathway [38]. Off-target effects were ruled out by repeating the animal experiments with a third, different region in the XMRV genome as a shRNA sequence.

No metastasis formation in lung, spleen and liver as well as bone metastasis was detected in control mice as well as shLTR mice (data not shown). Furthermore, the tumors did not vary in differentiation status, stromal cell organization nor did the tumors display differences in leukocyte infiltration, reflected by the relative presence of CD18^+^ leukocytes (data not shown). Interestingly, both set of xenograft experiments (shLTR1+2 as well as shLTR3) revealed reduced vascularization and increased areas of necrosis. Since we did not create a XMRV virus with a changed target site of the applied shRNAs that we could use to complement 22Rv1 cells transduced with shLTR3, it is possible that the observed in vivo and in vitro differences of these cells are a consequence of read through or recombinant XMRV and cellular transcripts.

Recent studies have suggested important functions of cytokines in various aspects of tumor growth. Most of the cytokines identified in our assays have been described to influence tumor microenvironment formation during prostate tumorigenesis. GRO/GROα (CXCL1), a member of the CXC chemokine family, promotes angiogenesis and recruits neutrophils and endothelial cells during malignant progression in prostate cancer. The aberrant expression of HGF (hepatocyte growth factor) and its receptor, c-Met, often correlate with advanced prostate cancer stages. Similarly, IGFBP2 and 4 (insulin like growth factor binding proteins 2 and 4) are biomarkers for advanced prostate cancer stages. Tissue inhibitors of matrixmetalloproteinases (TIMP) have independently of their function - inhibiting the proteinase activity of MMP (matrixmetalloproteinases) been described as factors involved in tumor progression: TIMP 1 and 2, both inhibit tumor cell apoptosis; TIMP 1 promotes tumor angiogenesis and TIMP 2 accelerates tumor progression.

XMRV-induced cytokine release does not appear to be tumor epithelial cell specific but can also be observed using prostate stromal fibroblasts (Figures 5; S4). We would like to point out that in these experiments XMRV viral stocks derived from LNCaP cells transfected with proviral XMRV VP62 DNA were used. We did not sequence the virus population derived from de novo infected LNCaP cells to rule out acutely transforming XMRV variants derived by recombination events as recently published [39].

XMRV infection of stromal fibroblast resulted in statistically significant increase of migrating LNCaP cells which was furthermore increased in cells infected for longer period of time (28d). This increase might be specific for XMRV and dependent on XMRV replication: a related MLV (MoMCF, an amphotropic MLV using PIT2 as receptor) did not result in higher amounts of LNCaP cells migrating (Figure 6B). Interestingly, supernatant from cell infected with replication defective XMRV-env pseudotyped particles did not result in changes of cell migration suggesting that receptor binding does not contribute to the observed changes.

In general, our observations are in concordance with some aspects of recently published data showing that XMRV infection of LNCaP cells promotes proliferation, transformation and invasiveness of these cells in vitro [40] as well as it was recently shown that XMRV infection can induce apoptosis in some human cell lines [41]. While we also observe an increase in invasiveness of cells when incubated with culture supernatant of XMRV infected cells, we did not observe an increase of proliferation due to XMRV infection, neither 22Rv1 cells transduced with shRNAs targeting XMRV transcripts (Figure S1) nor LNCaP cells or stromal cells infected with XMRV (data not shown) showed an decrease or increase in the proliferation rate.

We did not observe differences in MMP9 mRNA levels as a consequence of XMRV infection as recently published [40]; although a reduction of TIMP2 release of PrSc cells infected with XMRV was observed. Since MMP are mainly regulated on the protein levels we cannot exclude difference in MMP9 activity. Cytokine antibody arrays used here did not include MMP9 or MMP2 as well as we did not include zymography techniques to test for differences in MMP9 or MMP2 protein activity. It is understood that the rate and extent of angiogenesis is a critical component of tumor progression. The exact mechanism through which XMRV may affect angiogenesis, vessel formation and differences in cytokine release is not understood.

Retrovirus contamination seems to be frequent among widely used cell lines [11], [42], [43], [44], [45], [46], [47]. The unknown presence of retroviruses in cell lines beside the biohazard risk could affect the outcomes of experiments. Recent studies clearly demonstrate that human cell lines including prostate cancer cell lines commonly carry gammaretroviral sequences [6], [20], [48]. For some of them infectious particle formation has been demonstrated: human T-cell line Jurkat J6, lymphoblastoid cell line A3.0/F7 [47], B-cell line DG75 [45] and the prostate cancer cell lines LAPC4, VCaP and EKVX [6], [48]; however, in only few examples the consequence on experimental outcome has been demonstrated [44], [47], [49]. We conclude that experimental results obtained in vitro or in vivo conducted with retrovirus positive cell lines (in particular 22Rv1 or CWR-R1) might reflect molecular properties of the virus rather than cell type specific characteristics. Therefore, retroviral status of cell lines used in experiments should be provided as well as studies (including xenograft in vivo experiments) should be validated using multiple cell lines.

## Materials and Methods

### Cell Lines

The human prostate cancer cell lines LNCaP (ATCC #CRL-1740) and 22Rv1 (ATCC #CRL-2505) were grown in RPMI 1640 (Gibco) supplemented with 10% FCS, 5% Penicillin/Streptomycin and L-glutamine. Similar conditions were applied to shRNA treated 22Rv1 cells. TE671 (ATCC #CRL-8805) and 293T cells (ATCC #CRL-11268) were cultured in DMEM Glutamax (Gibco) supplemented with 10% FCS, 5% Penicillin/Streptomycin. HMEC cells (Lonza) were cultured in Endothelial Cell Growth Medium MV2 (PromoCell). Stromal cell lines (PrSc) were established as described [29], [50], [51]. Standard immunocytochemistry procedures together with FACS analysis were used to evaluate the stromal cell phenotype: negative cytokeratin expression was confirmed applying a pan-cytokeratin Ab (Santa Cruz Biotechnology, sc-8018) while vimentin expression was visualized using the mAb sc-7557 (Santa Cruz Biotechnology).

### Virus Infections

LNCaP cells chronically infected with XMRV (LNCaP i) were generated by transfection of XMRV VP62 proviral DNA [31]. Supernatant from XMRV producing cells (at least 80% confluence) was filtered (0.2 µm) and directly used in PrSc infection experiments: 2×10^5^ cells were infected using 1 ml infectious supernatant containing 8 µg/ml polybrene; cells were centrifuged 800×g for 1 h at 37°C. For the production of amphotropic Mo-MCF containing supernatant, 293T cells were transfected with proviral DNA (kindly provided by C. Stocking; [52] and chronically infected MoMCF 293T cells were cultured for several weeks.

### Production of env Pseudotyped Particles

Replication incompetent retroviral particles were produced as recently described [31]. Briefly, 5×10^6^ 293 cells were transfected with 5 µg pSF91-I-eGFP-PRE, 10 µg pSV-Mo-MLVgagpol and 5 µg of VP62 Env using CaPO4-HBS technique (Promega). Supernatant was passaged through a 0.2 µm pore size filter, aliquoted and frozen at −80C.

### Establishment of 22Rv1 XMRV Knock Down Cell Lines

shRNAs (sequences are listed in Table S1 and Figure 1) targeting two regions in the XMRV LTR (shLTR1 and shLTR2) were cloned in the lentiviral vector Lego G puro [27] using HpaI and XhoI restriction sites. Pseudotyped lentiviral supernatant containing Lego G puro shRNA was generated in 293 cells by co-transfecting expression plasmids encoding for HIV Rev, Gag/Pol and VSV-G Env [27]. Supernatant was harvested 72 h post transfection and immediately used for infection of 22Rv1 cells. After puromycin selection reduction of Gag protein levels were determined by Western blot and quantitative real-time RT-PCR. 22Rv1 control cells were infected with supernatant containing the parental Lego G puro plasmid. In addition, a third shRNA (shLTR3; see Table S1 and Figure 1) was used to control for shRNA off target effects. Proliferation of the cell lines was determined by MTT growth assay (Chemicon, Millipore).

### Western Blot Analysis

25 µg of total protein was separated by SDS PAGE and transferred on a PVDF membrane (Roth). Concentrated supernatant from the hybridoma cells CRL-1912 (ATCC) was used to detect XMRV p30-Gag protein. Equal protein amounts loaded were verified by incubation with anti-actin Ab 1501 (Chemicon).

### PCR/Real-time PCR

Total RNA was extracted using RNeasy extraction kit (Qiagen, CatNo.74104). 200 ng total RNA was DNaseI digested and subjected to RT-PCR using a random hexamer primer and SuperScript™ Reverse Transcriptase (Invitrogen, CatNo 18064-014). Nested XMRV gag PCR was performed (primer sequences are listed in Table S1) [8]. cDNA levels were quantified using a Qiagen Rotorgene Q 5plex instrument and Rotorgene 1.7 software as described previously [8], [18]. Two independent qPCR reactions were performed from two individually extracted RNA samples. Relative mRNA levels were normalized against GAPDH. Virus particles amounts were determined using 22Rv1 cells as a reference.

Expression levels of cytokines were analyzed accordingly; primer pairs are listed in Table S1. Relative mRNA levels were normalized to three housekeeping genes: GAPDH, RLP13 and TBP1. Cytokine expression levels illustrated in Figure 5 were normalized against three housekeeping genes and expressed as relative gene expression (ΔΔct) compared to mock infected cells at each individual time point. The experiment was performed three times in triplicates.

### Xenograft Mouse Experiments

Male *Mus musculus* SCID mice, 5–6 weeks of age, were purchased from Charles River Laboratories. Mice were kept under biosafety level 2 conditions in the animal facilities of the University Medical Center Hamburg-Eppendorf. Experiments were performed according to the national and international guidelines for care and use of experimental animals and were approved by the University’s ethical boards as well as German Animal Protection Law (No GV07/09). Xenograft tumors were generated under two-way conditions (22Rv1 control vs. 22Rv1 shXMRV in the presences of matrigel). Frozen aliquots of cells (4×10^6^) were thawed and suspended in 200 µl medium. Cells were mixed with 200 µl matrigel immediately prior injection. Using a 25-gauge needle, 200 µl of the cell-matrigel suspension were injected s.c. in each flank. On day 40, mice were sacrificed; tumors, lung, liver and spleen were surgically removed and incubated in 4% PFA/PBS overnight. Organs were embedded in paraffin. 5-µm sections were stained with H&E for histological analysis.

### Immunohistochemistry

Sections were deparaffinized using xylene and rehydrated using a series of graded ethanol. Sections were heated 4×2 min in citrate buffer using a microwave oven (650 W) and cooled down to room temperature. Blocking of non-specific binding sites was performed for 30 min at RT with 10% swine serum in antibody dilution buffer (Dako). Slides were blocked for endogenous biotin with avidin/biotin solution (Dako). CD34 antibody (Abcam 8158) was incubated for 2 h at RT followed by incubation with the secondary antibody (Dako E0468) for 30 min. Staining was performed with alkaline phosphatase solution (Dako, AK 5000) for 30 min at RT and IHC staining solution containing levamisole was added to the slides for 15–20 min. Samples were counterstained with Mayers hamin solution. Sections were coated with crystal mount mounting medium and embedded with Eukitt.

### Analysis of Necrotic Areas

Tumor sections were H&E stained and analyzed on an upright Zeiss Axioskop 2 plus microscope (Carl Zeiss, Jena, Germany), equipped with a Leica DFC 290 FX camera using HC PLAN S 10×/25 and N PLAN APO 5×/0.11 lenses. Images were acquired using Openlab software (Improvision, Coventry, United Kingdom) and Leica Application Suite software. Whole tumor section size and necrotic areas were calculated using Adobe Photoshop CS3 (Adobe Systems, San Diego, CA).

### Invasion Assay Using Matrigel™ Invasion Chambers (BD BioCoat™)

On day one, chamber inserts were rehydrated for two hours in RPMI. 1×10^5^ LNCaP cells were seeded. Day 2, 5×10^4^ PrSc cells infected or mock infected were seeded in the bottom compartment of the invasion chamber. Day 3, remaining LNCaP cells were removed from the insert using PBS and a cotton bud. Migrated cells were fixed with 8% PFA and stained with crystal violet. Number of migrated cells was determined by two different individuals counting three random fields. The arithmetic mean of three chambers was used to compare migration levels in one experiment. The experiment was repeated twice.

### Tube Formation

50 µl BD Matrigel™ (BD, Franklin Lakes, NJ USA) (1∶1 with serum-free medium) was carefully applied in a 96-well plate. Procedure was performed on ice and with pre-cooled laboratory instruments. After 30 min at 37°C, 2.5×10^4^ HMEC cells were seeded in 100 µl serum-free medium and 100 µl supernatant was added. Tube formation was documented after 5 h. Tube length was analyzed with Adobe Photoshop (Adobe Systems, San Diego, CA) ruler tool.

### Statistical Analysis

For all statistical analyses given here, two-tailed, two-sided Student’s *t*-tests were used (GraphPad Prism software). Values of *P*≤0.05 were considered as statistically significant.

## Supporting Information

Figure S1 **No differences in proliferation between 22Rv1control and 22Rv1 shLTR1+2 or 22Rv1 shLTR3 cells.** Proliferation of lentiviral transduced 22Rv1 cells followed by MTT assay. Filled circles 22Rv1 control cells, filled squares 22Rv1 shLTR1+2 cells and filled triangles 22Rv1 shLTR3 cells.
(TIF)

Figure S2 **22Rv1 control cells show significant less necrosis than XMRV knock down cells.** H&E staining of xenografted tumors induced by 22Rv1 control cells (A, C) show less necrotic areas compared to tumors induced by XMRV knock down cells 22Rv1 shLTR1+2 (B) and 22Rv1 shLTR3 (D).
(TIF)

Figure S3 **Cytokine antibody blots of supernatant from 22Rv1 control and 22Rv1 shLTR1+2 cells.** Supernatant conditioned for 24 h by 22Rv1 control and 22Rv1 shLTR1+2 cells was applied to the human cytokine antibody array. The cytokines indicated by arrows are those found to be different in the conditioned medium from these two cell lines.
(TIF)

Figure S4 **Cytokine antibody blots of supernatant from PrSc control and PrSc infected cells.** Supernatant from PrSc mock infected cells and PrSc XMRV infected cells was analyzed 72 h past infection by human cytokine antibody arrays (A) and (B). Differences in cytokine release between the cell lines are indicated by arrows.
(TIF)

Table S1 **Primer.**
(DOCX)
