# Supplementary figures and images for: XMRV Induces Cell Migration, Cytokine Expression and Tumor Angiogenesis: Are 22Rv1 Cells a Suitable Prostate Cancer Model?
Source: PLoS One. 2012 Jul 27;7(7):e42321. doi: 10.1371/journal.pone.0042321 (PMC3407105; doi:10.1371/journal.pone.0042321)

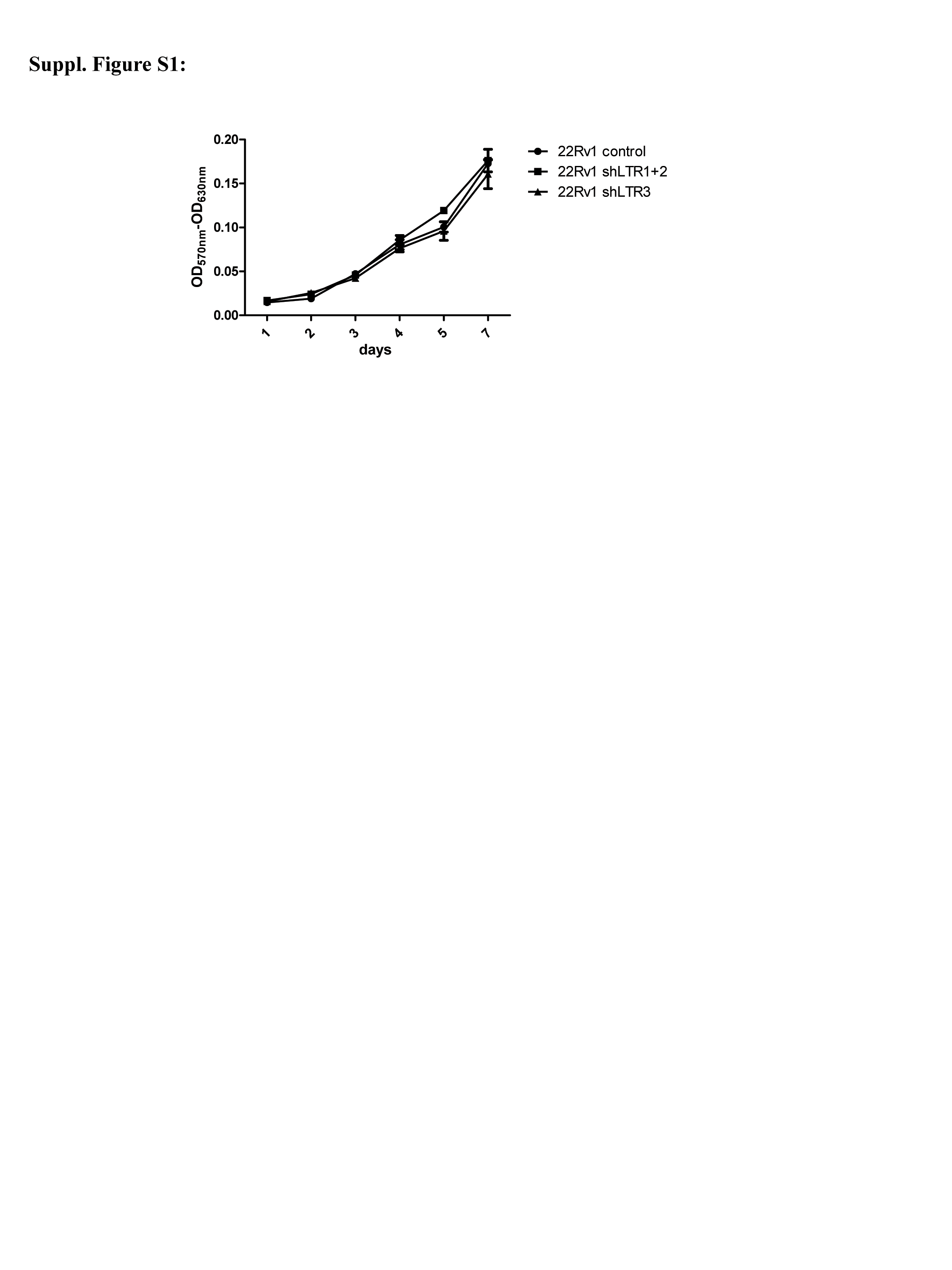

Supplement: Figure S1 — No differences in proliferation between 22Rv1control and 22Rv1 shLTR1+2 or 22Rv1 shLTR3 cells. Proliferation of lentiviral transduced 22Rv1 cells followed by MTT assay. Filled circles 22Rv1 control cells, filled squares 22Rv1 shLTR1+2 cells and filled triangles 22Rv1 shLTR3 cells. (TIF) [file pone.0042321.s001.tif]

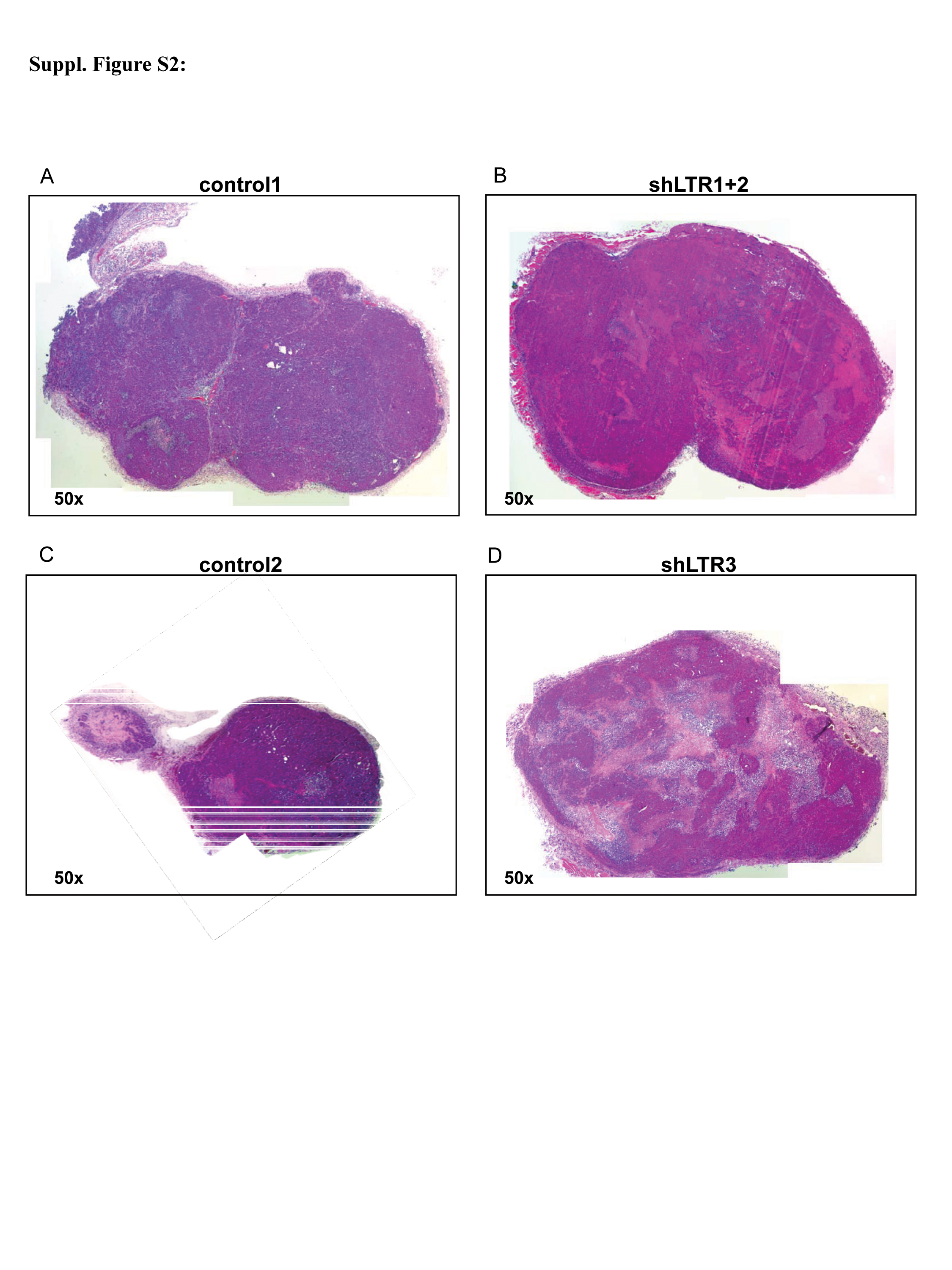

Supplement: Figure S2 — 22Rv1 control cells show significant less necrosis than XMRV knock down cells. H&E staining of xenografted tumors induced by 22Rv1 control cells (A, C) show less necrotic areas compared to tumors induced by XMRV knock down cells 22Rv1 shLTR1+2 (B) and 22Rv1 shLTR3 (D). (TIF) [file pone.0042321.s002.tif]

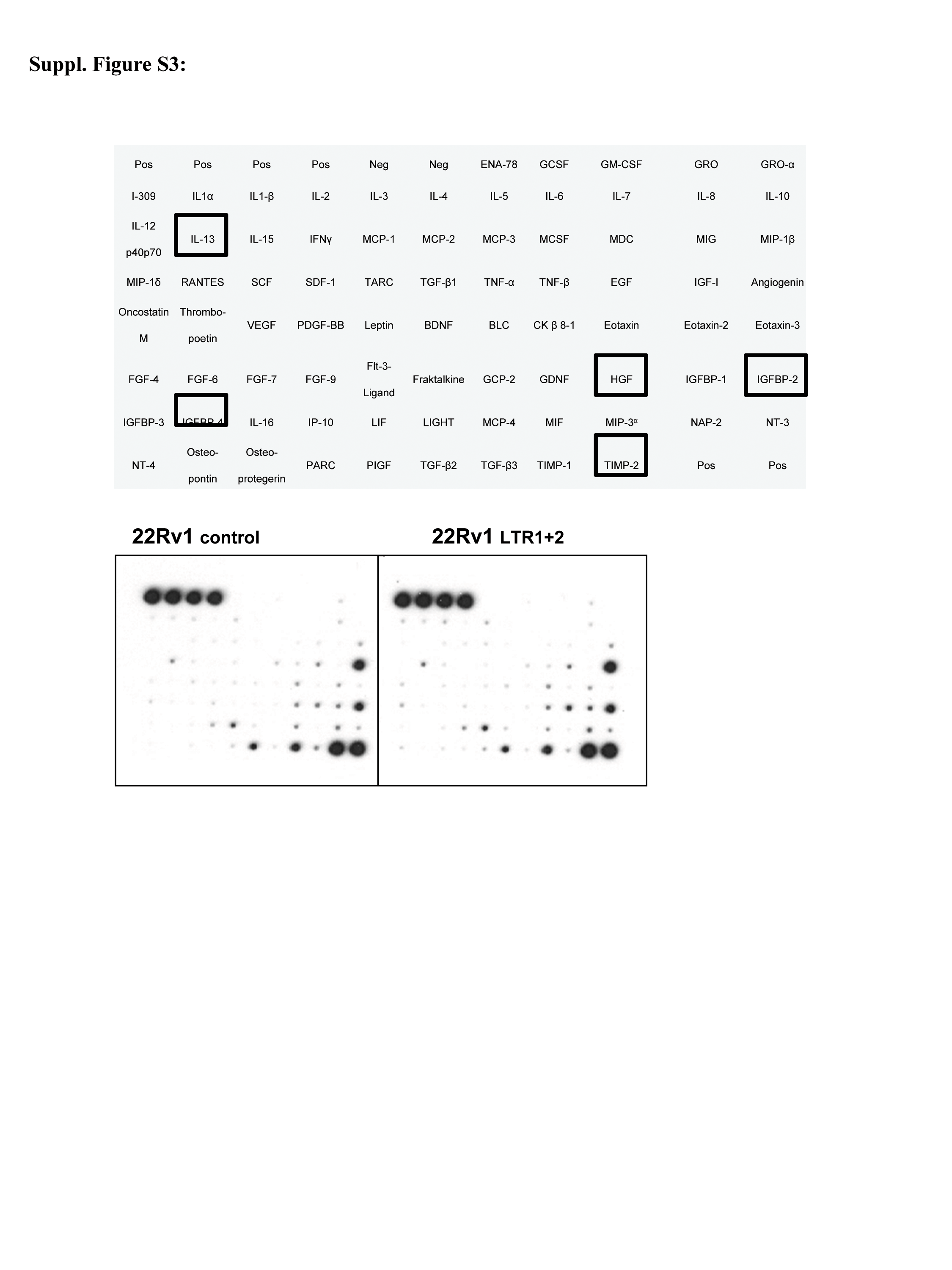

Supplement: Figure S3 — Cytokine antibody blots of supernatant from 22Rv1 control and 22Rv1 shLTR1+2 cells. Supernatant conditioned for 24 h by 22Rv1 control and 22Rv1 shLTR1+2 cells was applied to the human cytokine antibody array. The cytokines indicated by arrows are those found to be different in the conditioned medium from these two cell lines. (TIF) [file pone.0042321.s003.tif]

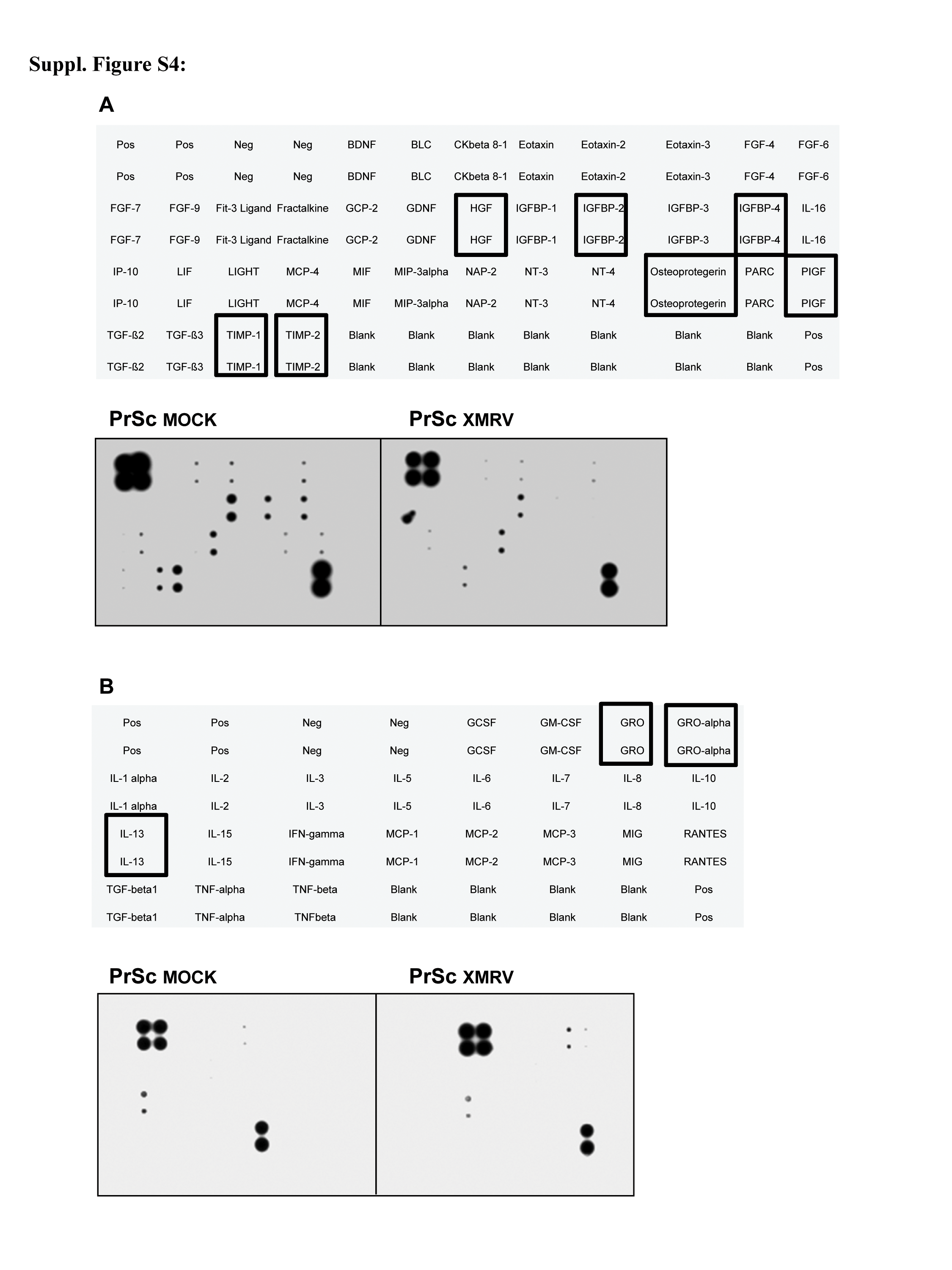

Supplement: Figure S4 — Cytokine antibody blots of supernatant from PrSc control and PrSc infected cells. Supernatant from PrSc mock infected cells and PrSc XMRV infected cells was analyzed 72 h past infection by human cytokine antibody arrays (A) and (B). Differences in cytokine release between the cell lines are indicated by arrows. (TIF) [file pone.0042321.s004.tif]
