# Supplementary material for: XMRV Induces Cell Migration, Cytokine Expression and Tumor Angiogenesis: Are 22Rv1 Cells a Suitable Prostate Cancer Model?
Source: PLoS One. 2012 Jul 27;7(7):e42321. doi: 10.1371/journal.pone.0042321 (PMC3407105; doi:10.1371/journal.pone.0042321)
Supplement: Supplementary file 2 [file pone.0042321.s005.docx]

name	primer (5‘-3‘)	PCR efficiency
**LTR 1**	F -AACGTTTGCTGTTTGCATCCGAAGCGTTCA<br>AGAGACGCTTCGGATGCAAACAGCAAATTTTTT-<br>R -TCGAGAAAAAATTTGCTGTTTGCATCCGA<br>AGCGTCTCTTGAACGCTTCGGATGCAAACAGCAAACGTT-	-
**LTR 2**	F -AACGTCTCCTCAGAGTGATTGACTACCTCAA<br>GAGGGTAGTCAATCACTCTGAAGGAGATTTTTTC-<br>R -TCGAGAAAAAATCTCCTCAGAGTGATTGAC<br>TACCCTCTTGAGGTAGTCAATCACTCTGAGGAGACGTT-	-
**LTR 3**	F -ACCGTGTCTTTGTCTCTGTCTTTGTGCTCAAG<br>AGGCACAAAGACAGAGACAAAGACATTTTTT-<br>R -TCGAGAAAAAATGTCTTTGTCTCTGTCTTTGT<br>GCCTCTTGAGCACAAAGACAGAGACAAAGACAC-	-
**RLP13A**	F -CTGGACCGTCTCAAGGTGTT-<br>R -GCCCCAGATAGGCAAACTT-	1.00
**GAG**<br><br><br><br>Real time	OF -CGCGTCTGAT TTGTTTTGTT-<br>OR -CCG CCTCTTCTTCATTGTTC-<br>IF –TCTCGAGATCATGGGACAGA-<br>IR –AGAGGGTAAGGGCAGGGTAA-<br>F- GGACTTTTTGGAGTGGCTTTGTT-<br>R- GCGTAAAACCGAAAGCAAAAAT-	-<br><br><br><br><br>1.15
**GAPDH**	OF –GAAGGTGAAGGTCGGAGTC-<br>OR -GAAGATGG TGATGGGATTTC	1.10
**TBP**	F -CCCATGACTCCCATGACC-<br>R -TTTACAACCAAGATTCACTGTGG-	1.02
**TIMP-1**	F -CACCAGAAGTCAACCAGACC-<br>R -AGTATCCGCAGACACTCTCC-	0.96
**TIMP-2**	F -TCCCTCCCTCAAAGACTGAC-<br>R -CCTACCTCCAAAATCCTTCCC-	0.94
**CXCL14**	F -CCCAAGCTGCAGAGCACC-<br>R -TAGACCCTGCGCTTCTCGTT-	1.08
**MMP9**	F -TGACAGCGACAAGAAGTG-<br>R -CAGTGAAGCGGTACATAGG-	1.17
**IL-13**	F -CAACTGAAACTTCGAAAGCATC-<br>R -ATTTTACCCCTCCCTAACCC-	1.13
**HGF**	F -GACCTACGAGAAAATTACTGCC-<br>R -TTTGCCATTCCCACGATAAC-	1.14
**GRO-a**	F -AGCGTCCGTGGCCACTGAAC-<br>R -CCATTCTTGAGTGTGGCTATG-	1.05
**IGFBP4**	F -GCCCAAGAGGACTGAGACTG-<br>R -CACATGCCAAAATCAGAGGA-	1.01
**OSTEOPONTIN (OPN)**	F -CTGACATCCAGTACCCTGATGC-<br>F -GGCCTTGTATGCACCATCA-	1.06
